# Functional analysis of *purM* in *Burkholderia cenocepacia* using a trimethoprim-selectable allelic exchange and mini-Tn7 complementation approach

**DOI:** 10.1128/spectrum.02492-25

**Published:** 2026-03-04

**Authors:** May Myat Noe, Kornvalee Meesilpavikkai, Yada Ajimathorn, Ajcharaporn Sawatpanich, Kritsakorn Saninjuk, Rasana W. Sermswan, Sunisa Chirakul

**Affiliations:** 1 Medical Sciences, Faculty of Medicine, Chulalongkorn University 26683, Bangkok, Thailand; 2 Center of Excellence in Immunology and Immune-mediated Diseases, Immunology Unit, Department of Microbiology, Faculty of Medicine, Chulalongkorn University 543913, Bangkok, Thailand; 3 Interdisciplinary Program of Medical Microbiology, Graduate School, Chulalongkorn University 214088, Bangkok, Thailand; 4 Division of Bacteriology, Department of Microbiology, Faculty of Medicine, Chulalongkorn University, King Chulalongkorn Memorial Hospital, Thai Red Cross Society 625869, Bangkok, Thailand; 5 School of Science, Mae Fah Luang University 295535, Chiang Rai, Thailand; 6 Microbial Products and Innovation Research Group, Mae Fah Luang University 68004, Chiang Rai, Thailand; 7 Department of Biochemistry, Faculty of Medicine, Khon Kaen University 26684, Khon Kaen, Thailand; 8 Center of Excellence in Antimicrobial Resistance and Stewardship, Faculty of Medicine, Chulalongkorn University 26683, Bangkok, Thailand; University of Dundee, Dundee, United Kingdom

**Keywords:** *Burkholderia cenocepacia*, *purM *gene, markerless gene deletion, mini-Tn7 complementation, trimethoprim-selectable system

## Abstract

*Burkholderia cenocepacia*, a member of the *Burkholderia cepacia* complex (Bcc) poses a significant threat to immunocompromised individuals, particularly those with cystic fibrosis. Progress in understanding its pathogenesis has been hindered by the availability of efficient genetic tools. We selected the *purM* gene for functional analysis because it is essential for growth under purine-limited conditions, intracellular survival, and virulence in the closely related species, *Burkholderia pseudomallei*. We adapted the non-replicative, trimethoprim-resistance (TMP^r^) vector pEDL1005 for markerless gene deletion in *B. cenocepacia* using an optimized *sacB*-based counterselection strategy, enabling in-frame deletion of *purM*, which encodes phosphoribosylaminoimidazole synthetase, in both the reference strain K56-2 and the clinical isolate SCBC075. Notably, efficient *sacB*-based counterselection required strain-specific sucrose concentrations (20% for K56-2 and 25% for SCBC075). To restore *purM* function, we employed a mini-Tn7-based complementation system (pUC18T-mini-Tn7T-TMP) for site-specific chromosomal insertion downstream of one of the four *glmS* homologs identified in this bacterium. A PCR-based method verified insertion 18–20 bp downstream of each target locus. Functional analysis revealed that *ΔpurM* mutants were strictly adenine auxotrophic, confirming the essential role of *purM* in *de novo* purine biosynthesis, consistent with findings in *B. pseudomallei*. However, unlike in *B. pseudomallei*, the Δ*purM* mutant of SCBC075 showed no significant attenuation in intracellular survival within RAW264.7 macrophages. These results validate pEDL1005 and the mini-Tn7 system as effective tools for unmarked gene deletion and chromosomal complementation in *B. cenocepacia*, expanding the molecular toolkit for this pathogen and enabling future studies of gene function, metabolism, and pathogenesis.

**IMPORTANCE:**

Genetic manipulation of *Burkholderia cenocepacia*, a challenging opportunistic pathogen, is essential for elucidating its pathogenesis. However, the available genetic tools remain limited. This study addresses this gap by adapting a pair of trimethoprim-selectable systems: the pEDL1005 vector for markerless gene deletion using strain-specific *sacB*-based counterselection, and a mini-Tn7-based strategy for site-specific chromosomal complementation. A key advancement is the development of a PCR-based method to verify precise transposon insertion downstream of *glmS*. Using these systems, we functionally characterized the *purM* gene, demonstrating that its deletion causes a purine-specific growth defect in minimal media. Together, these tools provide a robust platform for functional studies and enable future research into gene function and pathogenesis in this clinically relevant pathogen.

## INTRODUCTION

*Burkholderia* is a genus of obligately aerobic, gram-negative, non-spore-forming bacilli within the phylum Proteobacteria, order Burkholderiales, and family Burkholderiaceae (1). This genus includes the *Burkholderia cepacia* comple*x* (Bcc), a group of closely related environmental bacteria commonly found in soils, groundwater, and clinical settings (2). Among Bcc species, *Burkholderia cenocepacia* is a notable opportunistic pathogen capable of infecting both plants and humans (3, 4). However, several *Burkholderia* species are also known to engage in beneficial or synergistic interactions with plants, including nitrogen-fixing symbioses and plant-associated mutualism (5–7).

Clinically, *B. cenocepacia* poses a serious threat to immunocompromised individuals, particularly those with cystic fibrosis (CF) or chronic granulomatous disease (CGD) (8, 9). In susceptible patients, infection may lead to “cepacia syndrome,” a life-threatening condition characterized by high fever, progressive pulmonary deterioration, and sepsis (8). Despite its relatively low prevalence in certain regions such as Thailand (10), its multidrug resistance and capacity for nosocomial transmission present substantial therapeutic and epidemiological challenges (9, 11, 12).

Importantly, a key mechanism underlying persistence and treatment difficulty in chronic infection is its ability to survive and replicate within host immune cells, particularly macrophages, by actively delaying phagolysosome maturation (13). This intracellular survival is a critical determinant of its virulence and persistence in the host. A deeper understanding of the genetic basis of *B. cenocepacia* virulence and metabolism is therefore essential for the development of new therapeutic strategies. However, progress in dissecting the molecular mechanisms of its intracellular pathogenesis remains challenging primarily due to the limited availability of robust and versatile genetic tools. This limitation stems partly from the species’ large, multi-replicon genome structure and high antibiotic resistance profile (9).

Previous studies have employed allelic exchange systems based on suicide vectors carrying counter-selectable markers, such as *pheS*, *sacB*, and I-SceI, to generate markerless gene deletions for functional genetic analyses, including investigation of intracellular survival and virulence of pathogenic bacteria (14–18). Counterselection markers enable the selective elimination of cells retaining unwanted genetic material by imposing a lethal growth disadvantage under defined conditions, thereby enriching for correctly recombinant mutant (19). Markerless mutations are increasingly favored in microbial genetics because they eliminate residual resistance cassettes that may interfere with downstream genetic manipulation, cause polar effects on adjacent genes, or limit the number of selectable markers available for subsequent studies. Additionally, transposon-based systems have been utilized for insertional mutagenesis or chromosomal complementation (20, 21).

Nevertheless, none of the existing systems utilize a single trimethoprim-selectable marker for both scarless gene deletion and site-specific complementation, an approach that significantly simplifies genetic workflows in *B. cenocepacia* by reducing cloning complexity and avoiding the introduction of multiple resistance cassettes. Trimethoprim (TMP) is particularly advantageous in *B. cenocepacia* because very few antibiotics provide strong, reliable selection due to the organism’s extensive intrinsic resistance. In contrast, *B. cenocepacia* is highly susceptible to TMP, allowing robust positive selection. This makes TMP one of the few antibiotics that consistently yields clean, high-stringency selection during both allelic exchange and mini-Tn7-based complementation.

The *purM* gene encodes phosphoribosylaminoimidazole synthetase, an essential enzyme for *de novo* purine biosynthesis and thiamine metabolism (22, 23). In other bacterial species, including the closely related pathogen, *Burkholderia pseudomallei*, *purM* has been shown to be essential for growth under purine-limited conditions, intracellular survival, and virulence (24, 25). Purine limitation occurs in minimal or nutrient-restricted media and is also imposed during intracellular infection, where host cells restrict nucleotide availability as part of nutritional immunity. The strong requirement for *de novo* purine biosynthesis in *B. pseudomallei* is supported by the severe attenuation of Δ*purM* mutants in cell-based assays and animal models (24, 25), indicating that the host environment creates purine-restricted conditions that limit bacterial replication. However, the role of *purM* in *B. cenocepacia* has not been characterized.

In this study, we expanded the genetic toolkit for *B. cenocepacia* by adapting the TMP-resistance suicide vector pEDL1005, originally developed for *Burkholderia ubonensis* (26), for use in markerless gene deletion. We also employed the mini-Tn7 system (pUC18T-mini-Tn7T-TMP) for stable chromosomal complementation at the *att*Tn7 site downstream of *glmS* homologs and established a PCR-based strategy to verify the site-specific transposon insertion.

As a proof of concept, the previously uncharacterized *purM* gene was selected for targeted deletion and complementation. Assigning gene function in *B. cenocepacia* is complicated by its pronounced genomic and phenotypic heterogenicity. Therefore, a comparative, multi-strain approach was intentionally incorporated into our study design to identify potential strain-dependent differences in gene function and to ensure broader applicability of this genetic system.

To generate biologically robust and clinically relevant findings, we applied this genetic toolkit to two distinct strains: the well-characterized reference strain K56-2 and the clinically isolated strain SCBC075. Overall, this work provides a streamlined and versatile genetic manipulation platform for *B. cenocepacia*, enabling future studies on gene function, metabolism, and pathogenesis in this clinically important pathogen.

## MATERIALS AND METHODS

### Bacterial strains and growth conditions

All bacterial strains and plasmids used in this study are listed in Tables 1 and 2, respectively. Bacteria were cultured on Lennox LB broth or agar (Bio Basic Inc.). When necessary, 100 µg/mL trimethoprim (TMP) was used for transformant *E. coli* screening and to select the growth of *B. cenocepacia* merodiploid. Growth of the *B. cenocepacia* K56-2∆*purM* and SCBC075Δ*purM* mutants was supplemented with 40 µg/mL or 80 µg/mL adenine for broth or agar, respectively.

**TABLE 1:** Bacterial strains used in this study

Bacterial species	Strains	Characteristics	References
*Escherichia coli*	DH5a	Host for general cloning	(25)
	RHO3	Mobilizer strain used for conjugation	(26)
*Burkholderia cenocepacia*	K56-2	Wild-type (reference strain)	(22)
	K56-2::pEDL1005*ΔpurM*	Merodiploid strain	This study
	K56-2*ΔpurM*	*purM* knockout strain	This study
	SCBC075	Wild-type (clinical isolate)	This study (unpublished data)
	SCBC075::pEDL1005*ΔpurM*	Merodiploid strain	This study
	SCBC075*ΔpurM*	*purM* knockout strain	This study

**TABLE 2:** Plasmids used in this study^*a*^

Plasmid number	Plasmid name	Characteristics	Sources
	pEDL1005	TMP^r^; allelic exchange vector; sucrose or I-SceI counterselection	(21)
pPS1897	pUC18T-mini-Tn7T	TMP^r^; AMP^r^; pUC18T-mini-Tn7T-TMP	(27)
	pTNS3	Helper plasmid contains promoter P1 for transposase expression	(28)
pSC0061	pEDL1005*ΔpurM*_K56-2_	TMP^r^; pEDL1005 with 1154 bp Δ*purM*_K56-2_ (nt 4-1053 of 1056 nt ORF)	This study
pSC0064	pEDL1005D*purM*_SCBC075_	TMP^r^; pEDL1005 with 1174 bp Δ*purM_S_*_CBC075_ (nt 4-1053 of 1056 nt ORF)	This study
pSC0062	pUC18T-mini-Tn7T-*purM*_K56-2_	TMP^r^; pUC18T-mini-Tn7T-TMP with 1056 bp *purM* and 250 bp upstream intergenic region	This study

^*a*^ nt, nucleotide; ORF, open reading frame.

All experimental work involving *E. coli* or *Burkholderia cenocepacia* was conducted in a biosafety level 2 (BSL-2) laboratory in accordance with institutional biosafety guidelines. This study was reviewed and approved by the Faculty of Medicine, Chulalongkorn University Institutional Biosafety Committee (IBC approval no. MDCU-IBC012/2024). All personnel were trained in BSL-2 practices, and appropriate personal protective equipment and engineering controls were used throughout the study.

### Construction of *purM* deletion mutants

To assess the optimal sucrose concentration for *sacB*-based counter-selection, wild-type strains K56-2 (28) and SCBC075 were initiated from single colonies and grown overnight. These overnight cultures were subsequently streaked side-by-side onto YT agar plates containing varying sucrose concentrations, ranging from 15% to 30%. The plates were then incubated at 37°C for two days before being imaged.

*B. cenocepacia* strains K56-2 and SCBC075 were used as parental strains to generate in-frame deletion mutants of the *purM* gene (K56-2Δ*purM* and SCBC075Δ*purM*). The genomic organization, including flanking genes and their orientation, of the *purM* in *B. cenocepacia* strains K56-2 and SCBC075 is shown in Fig. S1. The upstream and downstream regions flanking *purM* were PCR-amplified using Q5 High-Fidelity DNA Polymerase (New England Biolabs) under the following conditions: initial denaturation at 98°C for 2 min; 35 cycles of 98°C for 1 min, annealing at 60°C for *B. cenocepacia* K56-2 or 64°C for SCBC075 for 30 s, and extension at 72°C for 1.5 min; followed by a final extension at 72°C for 7 min. PCR products were purified using the Zymo Research DNA Clean and Concentrator kit (Zymo Research). Gene replacement plasmids, pEDL1005∆*purM*_K56-2_ and pEDL1005Δ*purM*_SCBC075_, were constructed using Gibson assembly (27, 29). Overlapping sequences for the target plasmid (pEDL1005) and inserts (Δ*purM* or *purM*-Up and *purM*-Down) were designed using the NEBuilder assembly tool v.2.7.1 (New England BioLabs). Genomic DNA from *B. cenocepacia* strains K56-2 and SCBC075, isolated using the PureDireX genomic DNA isolation kit (Bio-Helix, Taiwan), served as templates for PCR amplification. Plasmid DNA was isolated using PureDireX miniPREP kit (Bio-Helix). All constructed plasmids were verified using Sanger sequencing at Bionics Co., Ltd. (Seoul, Korea). Primers were purchased from IDT Technologies (Singapore), and their sequences are provided in Table 3.

**TABLE 3:** List of primers used in *purM* gene deletion and complementation experiments

Primer number	Primer sequence (5′–3′)	Purpose
SC104	TCC CTA CCC GGG CGG CCG CCT CGA GCC GTC GGC ATG AGC GTT G	*purM*_K56-2_ deletion (Up)
SC105	TTC CCC CAT GTG ACG CAC TGC CTG CAG T	*purM*_K56-2_ deletion (Up)
SC106	CAG TGC GTC ACA TGG GGG AAT GCG AGA AG	*purM*_K56-2_ deletion (Down), *purM*_SCBC075_ deletion (Up)
SC107	GGG ATA ACA GGG TAA TCC CGA ATT CGC ACT TCG TTG AAC AGG TTG	*purM*_K56-2_ deletion (Down), *purM*_SCBC075_ deletion (Up)
SC205	TTC CCC CAT GTG ACG CAC GGC AAG CAG A	*purM*_SCBC075_ deletion (Down)
SC206	GGG ATA ACA GGG TAA TCC CGC GAG CAG AAA CCC GTG TAA TTG	*purM*_SCBC075_ deletion (Down)
SC121	CAT GCA TGA GCT CAC TAG TGA ACA GTC ACA ATG CGG GTC	*purM*_K56-2_ complementation
SC122	TTC GCG AGG TAC CGG GCC CAT CAG ACC ACG ACC GTC TG	*purM*_K56-2_ complementation

For construction of *B. cenocepacia* K56-2*ΔpurM*, which contains a 1,050 bp deletion in *purM*, 579 bp upstream and 649 bp downstream fragments of the *purM* gene (designated *purM*_K56-2_-Up and *purM*_K56-2_-Down) were PCR-amplified from *B. cenocepacia* K56-2 genomic DNA using primers SC104/SC105 and SC106/SC107, respectively. These fragments were assembled with XhoI- and EcoRI-HF-linearized pEDL1005 using NEBuilder HiFi DNA Assembly Master Mix (New England BioLabs), resulting in the gene replacement plasmid pEDL1005∆*purM*_K56-2_ (Fig. S2A).

For the construction of *B. cenocepacia* SCBC075*ΔpurM*, which contains a 1,050 bp deletion in *purM*, 652 bp upstream and 592 bp downstream fragments of the *purM* gene (designated *purM*_SCBC075_-Up and *purM*_SCBC075_-Down) were PCR-amplified from *B. cenocepacia* SCBC075 genomic DNA using primers SC107/SC106 and SC205/SC206, respectively. These fragments were assembled with XhoI- and EcoRI-HF-linearized pEDL1005 using NEBuilder HiFi DNA Assembly Master Mix (New England BioLabs), resulting in the gene replacement plasmid pEDL1005∆*purM*_SCBC075_ (Fig. S2B).

The plasmid-borne deletion constructs pEDL1005∆*purM*_K56-2_ and pEDL1005∆*purM*_SCBC075_ were first transformed into *E. coli* DH5α (30) and subsequently subcloned into *E. coli* mobilizer strain RHO3 (31). These plasmids were then transferred from *E. coli* RHO3 to *B. cenocepacia* strains K56-2 and SCBC075, respectively, as previously described (31). To identify merodiploids (*B. cenocepacia*::pEDL1005∆*purM*), we screened for blue colonies on LB agar containing 100 µg/mL TMP and 50 µg/mL X-Gluc (5-bromo-4-chloro-3-indolyl-beta-D-glucuronic acid). This color change confirmed integration of the pEDL1005Δ*purM* plasmid, as its *gusA* gene expresses beta-glucuronidase, which cleaves the X-Gluc substrate, yielding a blue product. At this stage, the chromosomally integrated merodiploid harbors the plasmid-borne *sacB* gene, enabling sucrose-based counterselection during resolution of the second crossover event. Merodiploids were subsequently resolved on YT agar supplemented with 20% sucrose for *B. cenocepacia* K56-2 or 25% sucrose for *B. cenocepacia* SCBC075, along with 50 µg/mL of X-Gluc, to select for second-crossover deletion mutants.

Deletion of *purM*, which confers purine auxotrophy, was preliminarily verified by patching white colonies (indicating loss of plasmid backbone) onto M9 minimal medium containing 20 mM glucose (M9G), and M9G supplemented with 80 µg/mL adenine and 0.0005% thiamine (M9GAT). Clones that failed to grow on M9G but grew on M9GAT were selected as candidate Δ*purM* mutants. The deletion of *purM* was subsequently confirmed by PCR and Sanger sequencing using primers SC104/SC107 or SC107/SC206 for *B. cenocepacia* K56-2 or SCBC075, respectively.

### Construction of the *B. cenocepacia* Δ*purM*_K56-2_ complemented mutant

Mutant complementation was achieved using the mini-Tn7 system, which enables stable, site-specific single-copy insertions at *glmS*-associated sites in bacterial genomes (32, 33). The delivery plasmid containing mini-Tn7 elements expressing the wild-type *purM* gene under its native promoter was constructed by Gibson assembly of PCR fragments amplified from *B. cenocepacia* K56-2 genomic DNA. Specifically, a 1,306 bp fragment comprising the full-length *purM* gene (1,056 bp) and its upstream intergenic region (250 bp) was amplified using primers SC121 and SC122. The PCR product was then ligated into BamHI-HF- and HindIII-HF-digested pUC18T-mini-Tn7T-TMP, generating pUC18T-mini-Tn7T-TMP::*purM*_K56-2_. The resulting construct was co-electroporated with the helper plasmid pTNS3, which encodes the Tn7 transposase, into the *B. cenocepacia* K56-2∆*purM* under the following electroporation conditions: 2,500 V, 25 µF, and 200 Ω. Transformants were selected on LB agar supplemented with 100 µg/mL TMP, and successful complementation was initially verified by restoration of growth in the absence of purine supplementation (prototrophy).

### Verification of mini-Tn7 insertion site in *B. cenocepacia* K56-2

To verify the chromosomal insertion site of the mini-Tn7 element, site-specific PCR was performed. Primers targeting the Tn7 left end (P_Tn7L_) and *B. cenocepacia*-specific primers annealing downstream of identified *glmS* loci (P_BCGLMS1R_ -P_BCGLMS4R_) were used (primer sequences listed in Table 4). PCR conditions were as follows: initial denaturation at 98°C for 2 min; 35 cycles of denaturation at 98°C for 1 min, annealing at 68°C for P_BCGLMS1R_ and P_BCGLMS4R_ or 66°C for P_BCGLMS2R_ and P_BCGLMS3R_ (30 s each), and extension at 72°C for 2.5 min (P_BCGLMS1R_ and P_BCGLMS4R_) or 2 min (P_BCGLMS2R_ and P_BCGLMS3R_); followed by a final extension at 72°C for 7 min. PCR products were analyzed using agarose gel electrophoresis to confirm insertion at predicted *attTn7* sites.

**TABLE 4:** Primers used for mapping of Tn7 insertion sites in *B. cenocepacia* K56-2

Primers number	Primer name	Sequence (5′-3′)	PCR product size (bp)	Source
SC30	P_Tn7L_	ATT AGC TTA CGA CGC TAC ACC C		(28)
SC256	P_BCGLMS1R_	GTT GCC GCC GAG ATC GAC GC	361	This study
SC260	P_BCGLMS2R_	GCA GAA GAT CGT GCT CGG CG	302	This study
SC258	P_BCGLMS3R_	CGT CCG GCA TCA CAT CAA GG	563	This study
SC262	P_BCGLMS4R_	GCA TGA TGC CCG AGC TGC TG	339	This study

### Growth analysis of *B*. *cenocepacia* K56-2, SCBC075, and derivative strains

*B. cenocepacia* K56-2 and SCBC075 wild-type strains, along with their derivatives Δ*purM* mutants, and a K56-2Δ*purM::purM* complemented strain, were grown overnight individually in LB broth at 37°C. Overnight cultures were then adjusted to an OD_600_ of 0.5–0.6 in LB medium. Two microliters of each adjusted culture were inoculated into 96-well round-bottom plates containing 200 µL of the following media: LB broth; LB broth supplemented with 40 µg/mL adenine; M9 minimal medium with 20 mM glucose (M9G); M9G supplemented with 40 µg/mL adenine (M9GA); M9G supplemented with 0.0005% thiamine (M9GT); and M9G supplemented with both adenine and thiamine (M9GAT). Growth was monitored by measuring OD_600_ at 1-hour intervals for 24 h using an Epoch 2 microplate spectrophotometer (BioTek).

### Intracellular survival assay of *B. cenocepacia* K56-2, SCBC075, and derivatives in RAW 264.7 murine macrophages

RAW 264.7 macrophages (ATCC TIB-71) were seeded in 24-well plates (Wuxi NEST Biotechnology Co., Ltd.) at a density of 5 × 10^5^ cells per well and incubated at 37°C with 5% CO_2_. Cells were then infected with *B. cenocepacia* K56-2 or SCBC075 wild-type strains, along with their derivatives, at a multiplicity of infection (MOI) of 10. After 2 h of incubation at 37°C, cells were washed twice with sterile phosphate-buffered saline (PBS) (Gibco) and treated with complete DMEM containing 250 µg/mL gentamicin and 500 µg/mL ceftazidime to kill extracellular bacteria. This time point was designated as 2 h post-infection (hpi).

To assess intracellular bacterial survival, infected cells were further incubated for 3, 8, and 24 h post-infection (hpi). At each time point, cells were washed with PBS and lysed with 0.1% Triton X-100. Serial dilutions of the lysates were plated on LB agar supplemented with adenine and incubated at 37°C for 48 h to enumerate bacterial colony-forming units (CFUs).

## RESULTS

### Successful deletion of the *purM* gene in *Burkholderia cenocepacia* using a sucrose-optimized pEDL1005 allelic exchange system

**Fig 1:**
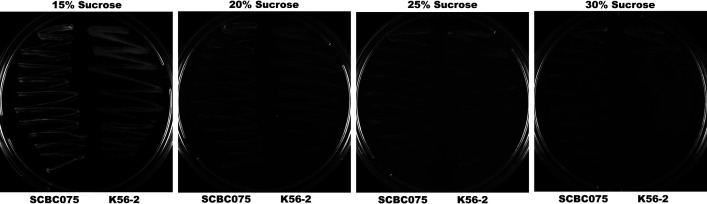
Optimization of sucrose concentrations for *sacB*-based counterselection in *B. cenocepacia* strains SCBC075 and K56-2. The growth of SCBC075 (left half of each plate) and K56-2 (right half) was assessed on YT agar supplemented with increasing concentrations of sucrose (15%, 20%, 25%, and 30%) to determine optimal concentrations for effective *sacB*-mediated counterselection during merodiploid resolution.

We successfully adapted a markerless allelic exchange system to generate in-frame deletion mutants of the *purM* gene in *B. cenocepacia* strains K56-2 and SCBC075 using the trimethoprim-resistance (TMP^r^), non-replicative gene replacement plasmid pEDL1005. This system employed *sacB*-based counter-selection using sucrose, replacing the I-SceI-based system previously applied in *B. ubonensis* (26). Unlike a prior report using *sacB*-mediated counter-selection in *B. pseudomallei* with 15% sucrose and the pEXKm5 plasmid (31), this sucrose concentration failed to yield any mutant clones in our experiments with *B. cenocepacia*. Therefore, we performed a sucrose optimization experiment. As shown in Fig. 1, both K56-2 and SCBC075 strains grew uninhibitedly in YT agar supplemented with 15% sucrose. However, 20% and 25% sucrose caused partial growth inhibition, while 30% resulted in nearly complete loss of viability, especially in K56-2. Based on these results, 20% sucrose was initially selected for counter-selection. At this concentration, K56-2 achieved 100% mutant recovery: all ten white colonies screened on YT agar supplemented with sucrose and X-Gluc showed the expected *purM* deletion phenotype. Specifically, mutants failed to grow on M9 minimal medium with 20 mM glucose (M9G) but grew on M9G supplemented with 80 µg/mL adenine and 0.0005% thiamine (M9GAT), confirming purine auxotrophy. In contrast, no SCBC075 mutants recovered at 20% sucrose. Increasing the sucrose concentration to 25% enabled recovery of a single mutant from seven screened colonies, indicating reduced efficiency. Deletion of the *purM* gene was confirmed by PCR and Sanger sequencing to ensure an in-frame deletion, excluding the START and STOP codons, thereby ruling out residual PurM activity.

### Complementation of the *purM* deletion mutant and characterization of *glmS*-associated insertion sites in *B. cenocepacia* K56-2

Complementation of the *purM* deletion mutant in *B. cenocepacia* K56-2, a purine auxotroph, was successfully achieved using the mini-Tn7 system (32). The *B. cenocepacia* K56-2*ΔpurM* mutant was transformed with pUC18T-mini-Tn7T-TMP-*purM*_K56-2_, which carries a 1,306 bp fragment comprising the intact *purM* gene (1,056 bp) and its upstream regulatory region (250 bp). Restoration of purine prototrophy in trimethoprim-resistant (TMP^r^) clones served as the initial screen for successful complementation. Colonies that grew on M9G medium lacking adenine were designated as *B. cenocepacia* K56-2D*purM::purM*_K56-2_.

To identify potential mini-Tn7 insertion sites, a genome-wide search was performed using the complete genome sequence of *B. cenocepacia* K56-2 (NCBI BioProject PRJNA627986) and the *Burkholderia* Genome Database (www.burkholderia.com) (34). Four *glmS* loci were identified: *glmS1* (K562_10640), *glmS2* (K562_20483), *glmS3* (K562_12462), and *glmS4* (K562_21244).

**Fig 2:**
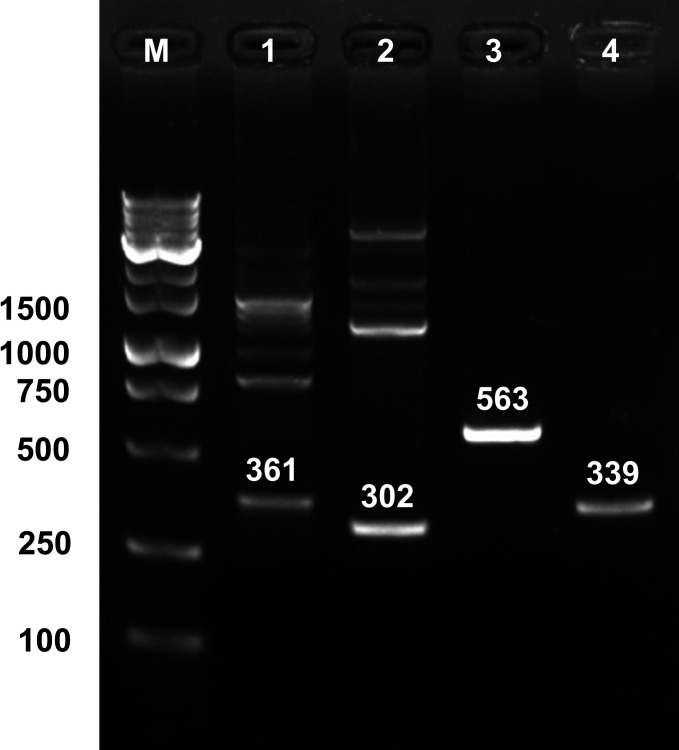
PCR analysis confirming site-specific mini-Tn7 integration at *glmS1-glmS4* loci in *B. cenocepacia* K56-2. Lane M: OneMARK B plus DNA ladder. Lanes 1–4: PCR products generated using primer P_Tn7L_ paired with locus-specific reverse primers, P_BCGLMS1R_ (lane 1, 361 bp), P_BCGLMS2R_ (lane 2, 302 bp), P_BCGLMS3R_ (lane 3, 563 bp), and P_BCGLMS4R_ (lane 4, 339 bp), indicating site-specific mini-Tn7 insertion downstream of the respective *glmS1-glmS4* loci. Electrophoresis was performed on a 2% agarose gel.

**Fig 3:**
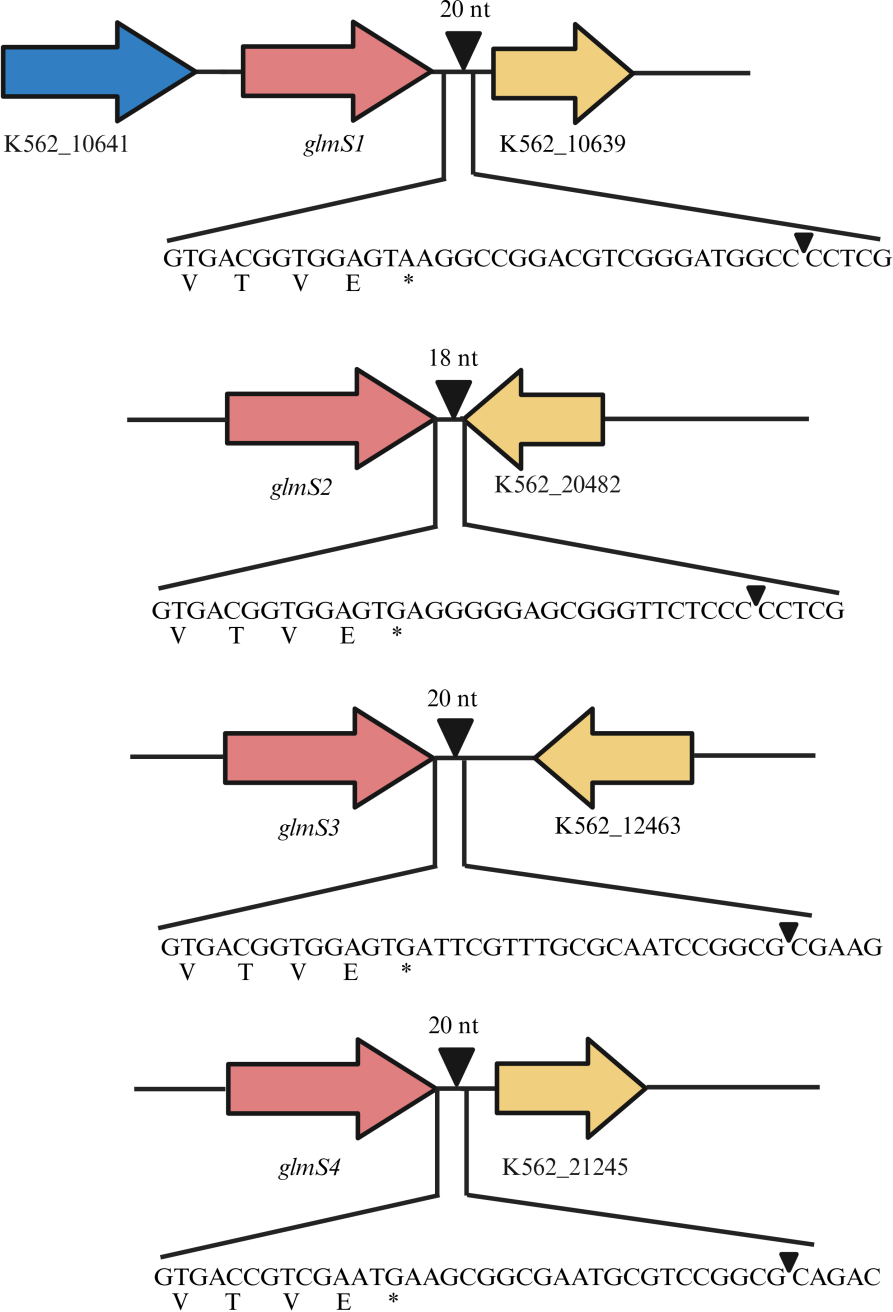
Mapping of mini-Tn7 insertion sites in *B. cenocepacia* K56-2. Putative Tn7 insertion sites (*att*Tn7) located downstream of *glmS1-glmS4* are indicated by black arrowheads. Arrowheads positioned above the nucleotide sequences represent experimentally confirmed mini-Tn7 insertion sites, as validated by PCR and sequencing. The adjacent downstream genes are labeled as K562_10639 (*glmS1*), K562_20482 (*glmS2*), K562_12463 (*glmS3*), and K562_21245 (*glmS4*). The nucleotide sequences at each insertion site are shown, with corresponding amino acid translations (VTVE*) to indicate the stop codon position.

To verify site-specific integration, eight TMP^r^ transformant colonies were selected for PCR screening. PCR was performed using the P_Tn7L_ primer paired with locus-specific reverse primers (P_BCGLMS1R_ - P_BCGLMS4R_), followed by Sanger sequencing. Amplification products of the expected sizes, 361 bp (*glmS1*), 302 bp (*glmS2*), 563 bp (*glmS3*), and 339 bp (*glmS4*), were obtained (Fig. 2). Each PCR product was generated only when the corresponding *glmS*-specific reverse primer was used, confirming site-specific integration. Sequence analysis of the PCR products revealed mini-Tn insertions 20 bp downstream of *glmS1* (*n* = 2), *glmS3* (*n* = 3), and *glmS4* (*n* = 2), and 18 bp downstream of *glmS2* (*n* = 1) (Fig. 3).

In summary, the *B. cenocepacia* K56-2*ΔpurM* mutant was successfully complemented via the site-specific integration of a single copy of the wild-type K56-2 *purM* gene using the mini-Tn7 system, with insertions confirmed to occur downstream of the *glmS* loci. Among the eight transformants analyzed, integration downstream of *glmS3* was observed most frequently (3 out of 8 colonies), suggesting that this locus may be a favorable site for transposon insertion in K56-2. Accordingly, the K56-2*ΔpurM::purM*_K56-2_ clone containing the *glmS3* insertion was selected for subsequent phenotypic and functional analyses. This observation may inform future studies employing the mini-Tn7 system in *B. cenocepacia*, particularly those aiming to compare gene function or expression from defined chromosomal sites.

### Deletion of *purM* confers adenine auxotrophy and impairs growth of *Burkholderia cenocepacia* K56-2 and SCBC075 in purine-limited media

**Fig 4:**
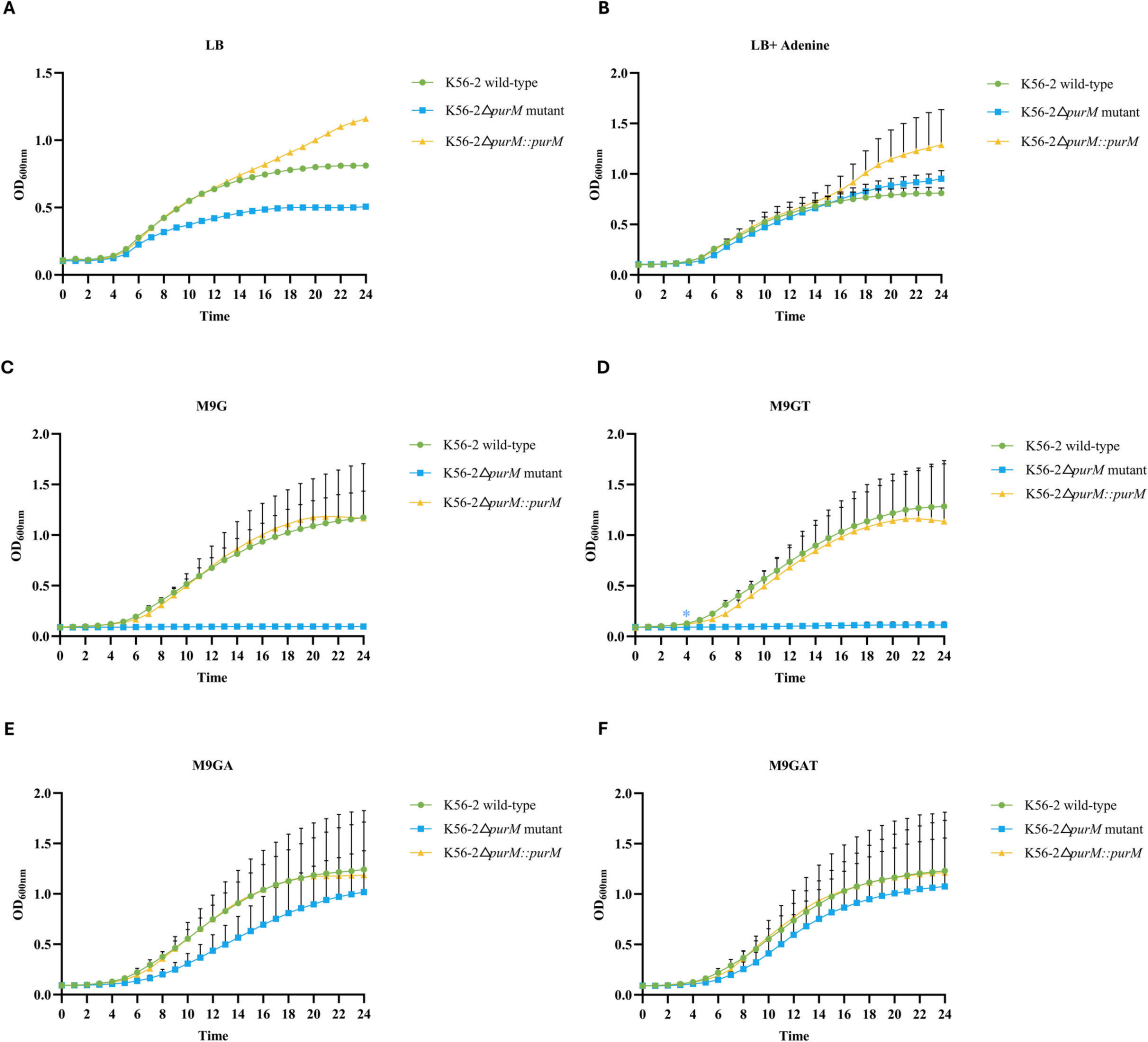
Growth curves of *B. cenocepacia* K56-2 strains in various media. The following strains were tested: K56-2 wild-type (green circles), K56-2Δ*purM* mutant (blue squares), and K56-2Δ*purM::purM*_K56-2_ complemented mutant (yellow triangles). Each strain was inoculated into 200 µL of the indicated media: LB broth (**A**), LB broth supplemented with 40 µg/mL adenine (**B**), M9 minimal medium with 20 mM glucose (M9G; **C**), M9G with 0.0005% thiamine (M9GT; **D**), M9G with 40 µg/mL adenine (M9GA; **E**), and M9G with both adenine and thiamine (M9GAT; **F**). Optical density at 600 nm (OD_600_) was measured hourly for 24 h. Error bars represent the standard deviation from three independent experiments.

We investigated the growth characteristics of *B. cenocepacia* K56-2 wild-type, K56-2Δ*purM*, and K56-2Δ*purM::purM* complemented strains in various media (Fig. 4). In nutrient-rich LB medium lacking adenine, the K56-2Δ*purM* mutant exhibited a reduced growth rate and lower final OD₆₀₀ compared to the K56-2 wild-type (Fig. 4A). However, when adenine was supplemented (LB + adenine), both strains showed comparable growth (Fig. 4B). In nutrient-restricted M9 minimal glucose medium (M9G), the K56-2Δ*purM* mutant failed to grow (Fig. 4C). Growth was markedly restored upon supplementation with adenine alone (M9GA; Fig. 4E) or in combination with thiamine (M9GAT; Fig. 4F), whereas thiamine alone had no effect (M9GT; Fig. 4D). Complementation with the wild-type K56-2 *purM* gene restored prototrophy, resulting in growth rates in M9G that were indistinguishable from those of the wild-type strain (Fig. 4A through F).

**Fig 5:**
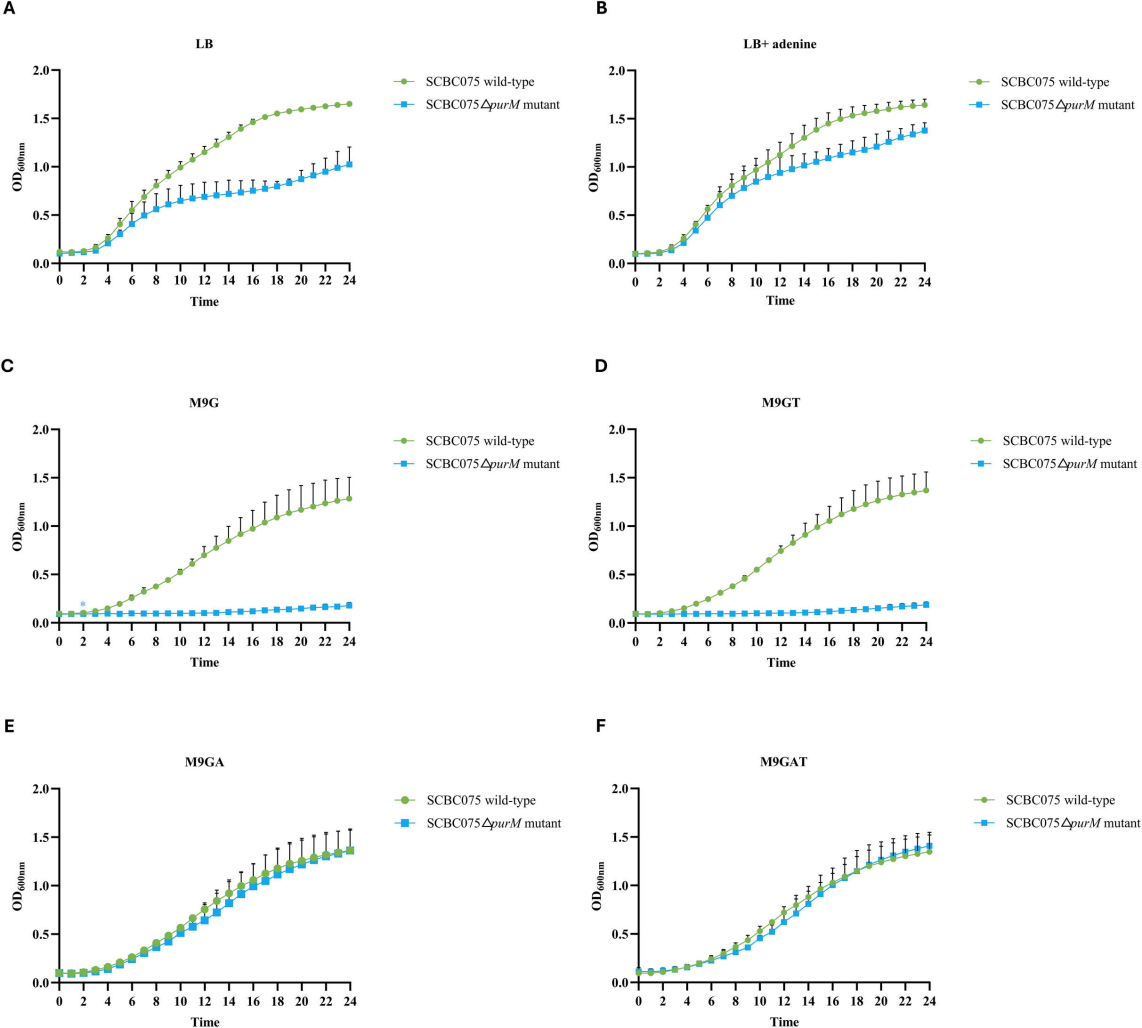
Growth curves of *B. cenocepacia* SCBC075 strains in various growth media. The following strains were tested: SCBC075 wild-type (green circles) and SCBC075Δ*purM* mutant (blue squares). Each strain was inoculated into 200 µL of the indicated media: LB broth (**A**), LB broth supplemented with 40 µg/mL adenine (**B**), M9 minimal medium with 20 mM glucose (M9G) (**C**), M9G with 40 µg/mL adenine (M9GA) (**D**), M9G with 0.0005% thiamine (M9GT) (**E**), and M9G with both adenine and thiamine (M9GAT) (**F**). Optical density at 600 nm (OD_600_) was measured hourly for 24 h. Error bars represent the standard deviation from three independent experiments.

Similarly, in LB medium, the SCBC075Δ*purM* mutant exhibited a reduced growth compared to the SCBC075 wild-type strain (Fig. 5A). Supplementation with adenine improved the growth of SCBC075Δ*purM* mutant, with OD₆₀₀ levels approaching those of the wild type (Fig. 5B). In M9G, the mutant failed to grow (Fig. 5C), but growth was restored upon supplementation with adenine alone (Fig. 5E) or in combination with thiamine (Fig. 5F), while thiamine alone had no effect (Fig. 5D), confirming a purine-specific growth defect.

These findings demonstrate that *purM* deletion results in strict adenine auxotrophy in *B. cenocepacia* and that *purM* is essential for growth under purine-limited conditions.

### Differential impact of *purM* deletion on intracellular survival of *Burkholderia cenocepacia* strains in RAW264.7 macrophages

To assess the impact of *purM* deletion on intracellular survival, RAW264.7 murine macrophage-like cells were infected with *B. cenocepacia* wild-type, Δ*purM* mutant, and Δ*purM::purM* complemented strains at a multiplicity of infection (MOI) of 10. Intracellular bacterial burdens were quantified at 3, 8, and 24 h post-infection (hpi) by lysing host cells and enumerating colony-forming units (CFUs) via serial dilution and plating.

**Fig 6:**
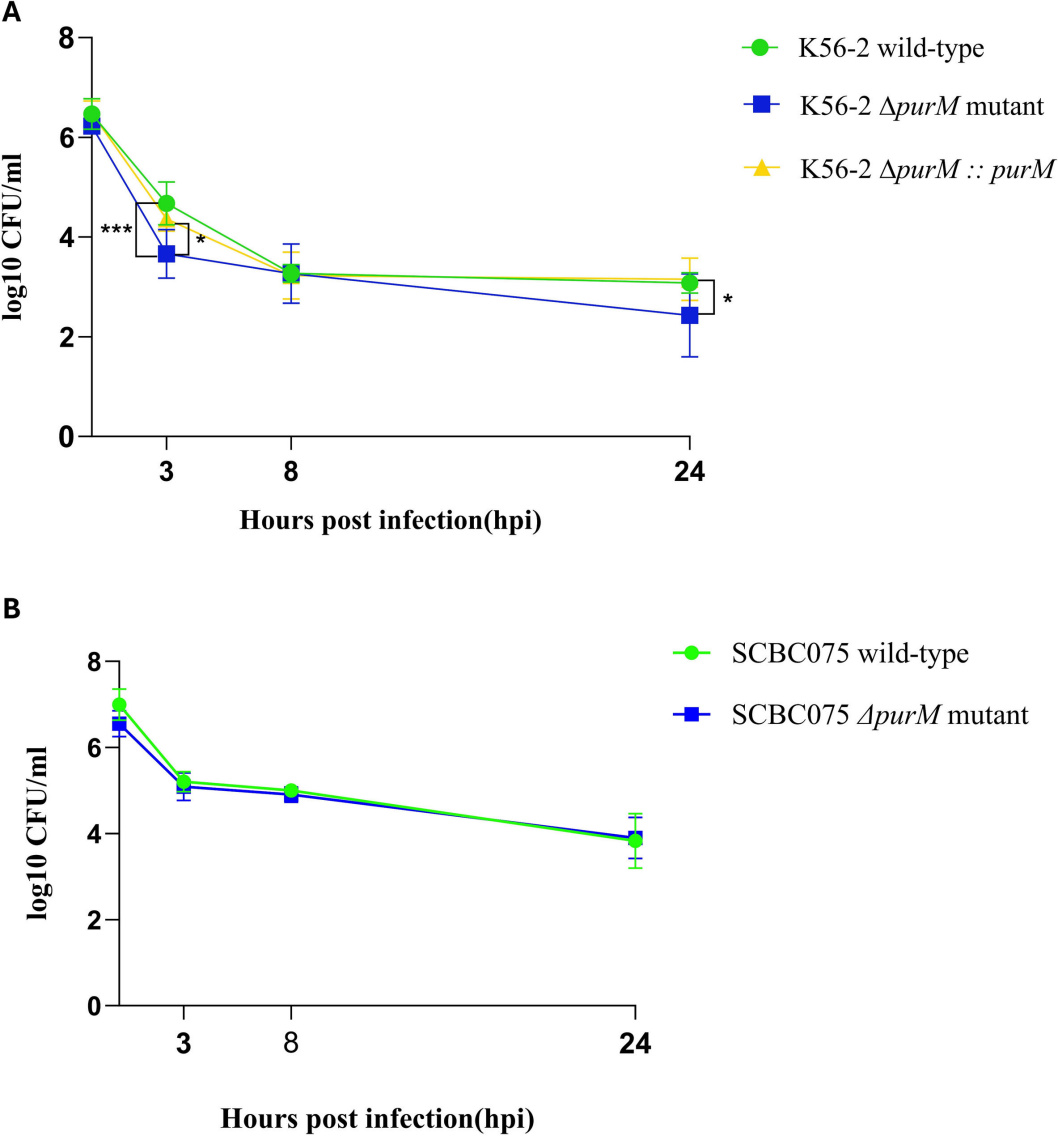
Intracellular survival of *B. cenocepacia* strains in RAW 264.7 cells. (**A**) RAW264.7 macrophage-like cells were infected with *B. cenocepacia* K56-2 wild-type (green circles), K56-2Δ*purM* mutant (blue squares), and K56-2Δ*purM::purM*_K56-2_ complemented strain (yellow triangles) at a multiplicity of infection (MOI) of 10. (**B**) RAW264.7 cells were infected with *B. cenocepacia* SCBC075 wild type (green circles) and SCBC075Δ*purM* mutant (blue squares), also at an MOI of 10. Intracellular bacterial survivals were determined at 3, 8, and 24 h post-infection (hpi). At each point, host cells were lysed, and intracellular bacteria were enumerated by serial dilution and plating. Colony-forming unit (CFU) counts are presented as log10 CFU/mL and represent the means of three independent experiments, with duplicate wells sampled per time point. Statistical significance was determined by two-way ANOVA for the K56-2 and by unpaired *t*-test for SCBC075 strains. Significance is indicated as follows: *P* < 0.05 (*) and *P* < 0.0005 (***).

At 3 hpi, the K56-2Δ*purM* mutant exhibited significantly reduced intracellular survival compared to the K56-2 wild type (*P* < 0.0005), indicating a defect in early intracellular adaptation or entry. By 8 hpi, CFU levels of the mutant recovered to those of the wild type. At 24 hpi, the mutant again displayed a statistically significant reduction in CFU counts (*P* < 0.05). The complemented strain (K56-2Δ*purM::purM*) consistently mirrored the wild type at all time points, confirming successful functional restoration of *purM* (Fig. 6A).

In the clinical isolate SCBC075, deletion of *purM* had no observable effect on intracellular survival. The SCBC075Δ*purM* mutant displayed CFU counts comparable to the SCBC075 wild type at all time points (Fig. 6B).

These findings reveal a strain-dependent role for *purM* in intracellular survival. While *purM* is critical for early adaptation of the reference strain K56-2 within macrophages, it appears dispensable in the SCBC075 clinical isolate. This suggests differential reliance on purine biosynthesis for intracellular survival among *B. cenocepacia* strains, possibly reflecting metabolic flexibility or strain-specific host interaction strategies.

## DISCUSSION

*Burkholderia cenocepacia* is a clinically significant member of the *Burkholderia cepacia* complex (Bcc), known for its high intrinsic antibiotic resistance and its ability to cause severe infections in immunocompromised individuals, particularly those with cystic fibrosis or chronic granulomatous disease (10, 12, 35). Its ability to survive in diverse environments and evade host immune responses further contributes to the difficulty of studying and treating this pathogen (10, 12, 36). Despite its clinical relevance, progress in elucidating the genetic and metabolic determinants of pathogenesis has been hindered by the limited availability of efficient and flexible genetic tools.

The *purM* gene encodes phosphoribosylaminoimidazole synthetase, a key enzyme in *de novo* purine and thiamine biosynthesis (22, 37). Although *purM* is not classically considered a virulence gene, its role in nucleotide metabolism suggests an important contribution to bacterial fitness under nutrient-restricted conditions (37). Consistent with findings in *Burkholderia pseudomallei*, where *purM* deletion impairs growth in minimal media and attenuates virulence in animal models (24, 25), our results confirm that *purM* is essential for purine biosynthesis in *B. cenocepacia*.

Notably, the phenotypic consequences of *purM* deletion differed between strains. While adenine supplementation fully restored growth in SCBC075Δ*purM*, K56-2Δ*purM* exhibited delayed recovery despite supplementation with both adenine and thiamine. These differences suggest strain-specific variation in purine salvage capacity, transport efficiency, or broader metabolic networks. Similar strain-dependent variability has been reported in *B. pseudomallei*, reflecting distinct ecological adaptations and evolutionary histories (24, 25).

Strain-dependent effects were also evident in intracellular survival assays. Deletion of *purM* in K56-2 significantly impaired early survival and persistence in murine macrophages, indicating that *de novo* purine biosynthesis contributes to fitness within the intracellular environment. In contrast, SCBC075Δ*purM* retained full intracellular competence despite exhibiting the same adenine auxotrophy. This divergence suggests that SCBC075 may rely more effectively on host-derived purines or possess more efficient salvage pathways that mitigate the loss of *purM* during infection. Together, these findings underscore that metabolic determinants of virulence can be highly context- and strain-specific, even within a single Bcc species.

Importantly, strain-specific differences were also evident at the level of genetic manipulation. Although the *sacB*-based counterselection system encoded on pEDL1005 enabled efficient allelic exchange in both strains, successful mutant recovery required empirical optimization of sucrose concentrations, with 20% sucrose optimal for K56-2 and 25% required for SCBC075. This observation highlights that, even within a single *B. cenocepacia* species, physiological heterogeneity can substantially influence the performance of commonly used counterselectable systems. Our findings reinforce prior reports in other *Burkholderia* species showing that *sacB*-mediated counterselection is highly strain-dependent and emphasize that genetic frameworks intended for broad use must accommodate such variability through strain-aware optimization rather than fixed protocols (38).

To restore *purM* function in K56-2, we employed a mini-Tn7-based chromosomal complementation strategy. Beyond functional restoration, this approach enabled systematic evaluation of *glmS*-associated *att*Tn7 insertion sites in the K56-2 genome. Although all four identified *glmS* loci supported mini-Tn7 insertion, integrations were most frequently recovered downstream of *glmS3*, suggesting that this site represents a favorable genomic landing pad for stable, single-copy complementation in *B. cenocepacia*. This pattern contrasts with *Burkholderia mallei*, where *glmS1* has been reported as the predominant mini-Tn7 insertion hotspot (32). Furthermore, the availability of multiple *glmS* genes does not always guarantee multiple functional *att*Tn7 sites. For instance, in *Acinetobacter baumannii*, despite the presence of two *glmS* genes, insertions occur exclusively downstream of *glmS2* due to the absence of the *att*Tn7 recognition sequence near *glm*S1 (39). In contrast, our findings confirm that all four *glmS* loci in *B. cenocepacia* remain viable targets, though they exhibit distinct hierarchy in usage. Notably, this conclusion is based on a limited number of screened colonies (*n* = 8), and additional sampling will be required to fully assess relative insertion biases among *glmS* sites.

A practical advantage of our genetic framework is the use of trimethoprim (TMP) as a single selectable marker for both allelic exchange and chromosomal complementation. In contrast to existing *Burkholderia* genetic systems that require multiple antibiotic markers, a unified TMP-selectable workflow reduces cloning complexity and avoids marker accumulation. Given the limited repertoire of antibiotics suitable for selection in *B. cenocepacia*, this streamlined approach provides a robust and practical tool for routine genetic manipulation.

In summary, this study establishes *purM* as a key metabolic determinant in *B. cenocepacia*, with strain-specific effects on growth and intracellular fitness that highlight pronounced physiological heterogeneity within the species. By integrating allelic exchange and site-specific complementation into a single TMP-selectable toolkit, our work expands the molecular genetic capacity available for studying metabolism, fitness, and virulence in this intrinsically drug-resistant pathogen and provides a framework adaptable to other members of the Bcc and similarly challenging bacterial species.

## SUPPLEMENTAL MATERIAL

Figure S1 spectrum.02492-25-s0001.tif Genomic organization of *purM* and its flanking genes in *B. cenocepacia* strains K56-2 and SCBC075.

Figure S2 spectrum.02492-25-s0002.tif Construction of gene replacement plasmids for *purM* deletion.

Supplemental legends spectrum.02492-25-s0003.docx Descriptive legends for Fig. S1 and S2.

ASM does not own the copyrights to Supplemental Material that may be linked to, or accessed through, an article. The authors have granted ASM a non-exclusive, world-wide license to publish the Supplemental Material files. Please contact the corresponding author directly for reuse.
