# Supplementary figures and images for: Functional analysis of purM in Burkholderia cenocepacia using a trimethoprim-selectable allelic exchange and mini-Tn7 complementation approach
Source: Microbiol Spectr. 2026 Mar 4;14(4):e02492-25. doi: 10.1128/spectrum.02492-25 (PMC13055311; doi:10.1128/spectrum.02492-25)

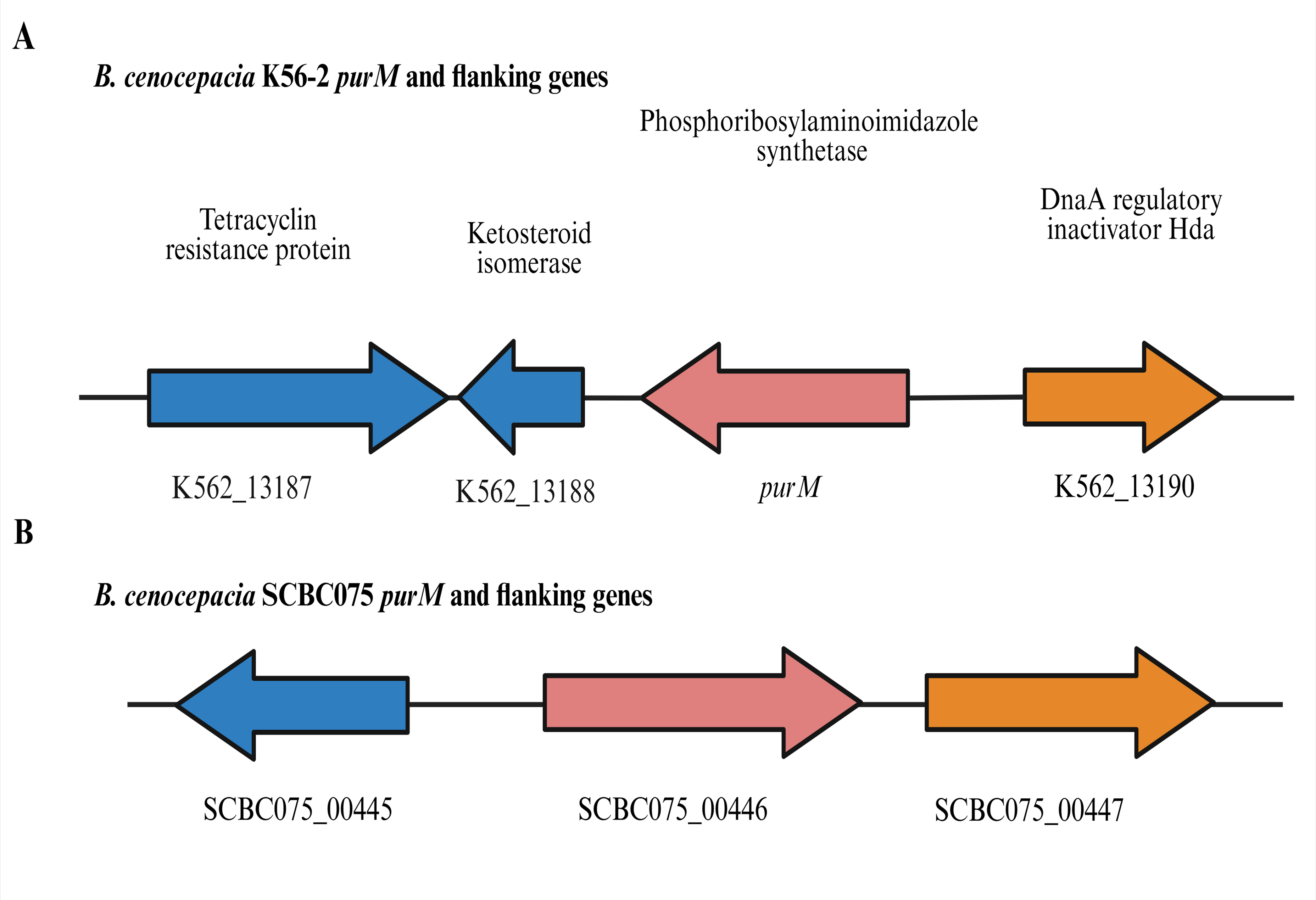

Supplement: Figure S1 — Genomic organization of purM and its flanking genes in B. cenocepacia strains K56-2 and SCBC075. [file spectrum.02492-25-s0001.tif]

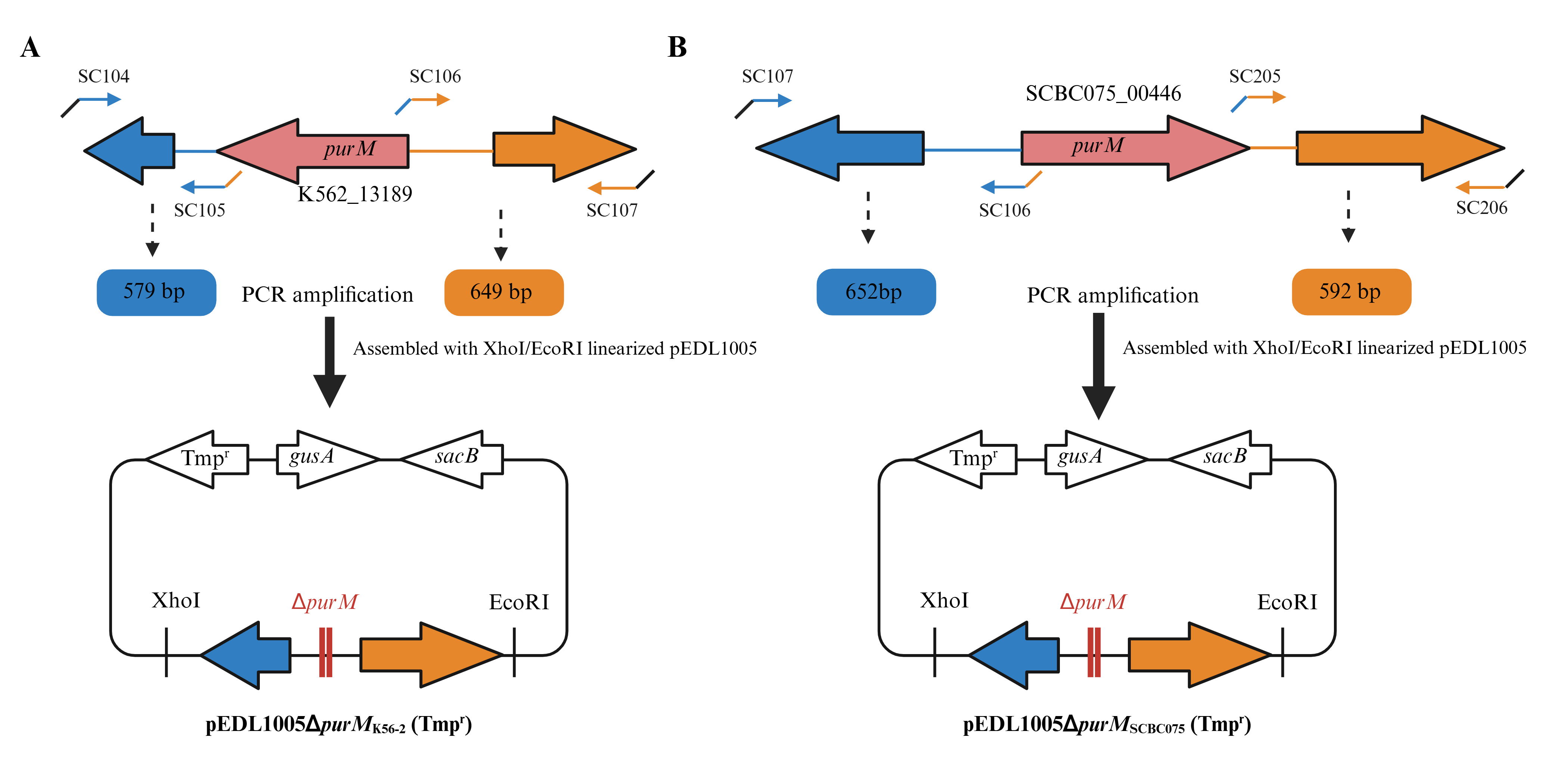

Supplement: Figure S2 — Construction of gene replacement plasmids for purM deletion. [file spectrum.02492-25-s0002.tif]
