# Supplementary material for: Functional analysis of purM in Burkholderia cenocepacia using a trimethoprim-selectable allelic exchange and mini-Tn7 complementation approach
Source: Microbiol Spectr. 2026 Mar 4;14(4):e02492-25. doi: 10.1128/spectrum.02492-25 (PMC13055311; doi:10.1128/spectrum.02492-25)
Supplement: Supplemental legends — Descriptive legends for Fig. S1 and S2. [file spectrum.02492-25-s0003.docx]

**[Supplemental material](https://journals.asm.org/writing-your-paper#supplemental-material)s**

**Figure S1. Genomic organization of purM and its flanking genes in B. cenocepacia strains K56-2 and SCBC075.**

The purM gene, encoding phosphoribosylaminoimidazole synthetase (pink), is flanked by upstream gene(s) (blue) and downstream gene (orange). Color coding indicates relative genomic position rather than functional similarity between strains. (A) In B. cenocepacia K56-2, the upstream genes are K562_13187, encoding a tetracycline resistance protein, and K562_13188, encoding a ketosteroid isomerase. The downstream gene is K562_13190, encoding DnaA regulatory inactivator Hda. (B) In B. cenocepacia SCBC075, the upstream gene is SCBC075_0045 and the downstream gene is SCBC_0047.

**Figure S2.** **Construction of gene replacement plasmids for *purM* deletion.** Schematic representation of the strategy used to construct pEDL1005Δ*purM* plasmids for *B. cenocepacia* K56-2 (A) and *B. cenocepacia* SCBC075 (B). The *purM* gene (pink) was targeted for deletion by replacing it with upstream (blue) and downstream (orange) flanking regions. For K56-2 (A), the upstream and downstream regions (579 bp and 649 bp, respectively) were amplified using primers SC104/SC105 and SC106/SC107. For SCBC075 (B), the flanking regions (652 bp and 592 bp, respectively) were amplified using primers SC107/SC106 and SC205/SC206. Each pair of PCR products was assembled into the XhoI- and EcoRI-HF- linearized pEDL1005 plasmid to generate the gene deletion constructs pEDL1005Δ*purM*_K56-2_ and pEDL1005Δ*purM*_SCBC075_.
